# Novel theory and potential applications of central diastolic pressure decay time constant

**DOI:** 10.1038/s41598-024-56137-8

**Published:** 2024-03-11

**Authors:** Vasiliki Bikia, Patrick Segers, Georgios Rovas, Sokratis Anagnostopoulos, Nikolaos Stergiopulos

**Affiliations:** 1 Laboratory of Hemodynamics and Cardiovascular Technology, Institute of Bioengineering, Swiss Federal Institute of Technology, EPFL STI IBI-STI LHTC, MED 3 2922 (Batiment MED), Station 9, 1015 Lausanne, Switzerland; 2 IBiTech, University of Ghent, Ghent, Belgium

**Keywords:** Central blood pressure, Vascular age, Asklepios study, Windkessel effect, Arterial pulse wave, Biomedical engineering, Diagnostic markers

## Abstract

Central aortic diastolic pressure decay time constant (${\uptau }$) is according to the two-element Windkessel model equal to the product of total peripheral resistance ($R$) times total arterial compliance ($C$). As such, it is related to arterial stiffness, which has considerable pathophysiological relevance in the assessment of vascular health. This study aimed to investigate the relationship of the constant ${\uptau }$ with the product $T\frac{MBP}{{cPP}}$, given by heart period ($T$) times the ratio of mean blood pressure (MBP) to central pulse pressure ($cPP$). The relationship was derived by performing linear fitting on an in silico population of n_1_ = 3818 virtual subjects, and was subsequently evaluated on in vivo data (n_2_ = 2263) from the large Asklepios study. The resulted expression was found to be ${\uptau } = k^{\prime}T\frac{MBP}{{cPP}},$ with $k^{\prime} = 0.7$ (R^2^ = 0.9). The evaluation of the equation on the in vivo human data reported high agreement between the estimated and reference ${\uptau }$ values, with a correlation coefficient equal to 0.94 and a normalized RMSE equal to 5.5%. Moreover, the analysis provided evidence that the coefficient $k^{\prime}$ is age- and gender-independent. The proposed formula provides novel theoretical insights in the relationship between ${\uptau }$ and central blood pressure features. In addition, it may allow for the evaluation of ${\uptau }$ without the need for acquiring the entire central blood pressure wave, especially when an approximation of the $cPP$ is feasible. This study adds to the current literature by contributing to the accessibility of an additional biomarker, such as the central diastolic pressure decay time constant, for the improved assessment of vascular ageing.

## Introduction

The shape of the arterial blood pressure pulse alters significantly during its transmission across the vasculature. In addition to the variation of systolic pressure characteristics, the diastolic pressure decay is inherently different across individuals, with no evidence of it being a monotonic function of time^1,2^. The central diastolic pressure decay is characterized by a time constant, ${\uptau }$, which has been used previously to describe the arterial Windkessel properties^3^. According to the two-element Windkessel model, ${\uptau }$ is equal to the product of the peripheral resistance ($R$) and the total arterial compliance ($C$). The time constant ${\uptau }$ can be calculated from the blood pressure waveform by fitting a mono-exponential decay function to the diastolic part of the curve.

Given the dependency of the central blood pressure waveform on structural and functional changes in the cardiovascular system, the diastolic pressure decay is also sensitive to the properties of the aortic wall and the peripheral vasculature, while it is influenced by factors such as age and blood pressure^3–5^. The constant ${\uptau }$ may therefore be an important metric in the assessment of cardiovascular function. Concurrently, there is emerging evidence on its utility in the diagnosis and management of various cardiovascular diseases^4,6–8^. Experiments in dogs showed that the time constant (${\uptau }$) of diastolic pressure decay correlated with variations in peripheral vascular resistance, indicating its practical value for continuous monitoring of systemic resistance. Moreover, it was found that aortic stiffening in hypertensive patients could impair myocardial viability by accelerating the diastolic exponential decay of central blood pressure, increasing the risk of ischemic heart disease. In another instance, it was observed that carotid ${\uptau }$ was higher than radial ${\uptau }$, and the two variables were not correlated, suggesting that local factors significantly influence diastolic pressure decay properties, making a systemic ${\uptau }$ value unreliable without modification. In addition, the concept of the diastolic pressure decay time constant has been widely exploited for the derivation of total arterial compliance^9^. Therefore, understanding and improving accessibility to this parameter could provide valuable insights into the main parameters defining the hemodynamics of the circulatory system and may add to the current assessment of vascular ageing, vascular stiffening, and the associated risk profile.

In this study, we derived an empirical relation between the central diastolic pressure decay time constant (${\uptau }$) (dependent variable), the duration of the heart period ($T$), and the ratio of mean blood pressure over central pulse pressure, namely the difference between central systolic and diastolic pressure, (independent variables). A linear relationship to estimate $\tau$ was derived by implementing linear fitting on previously published in silico data (n_1_ = 3818)^10^, which were generated using a well validated numerical model of the cardiovascular system^11^. Subsequently, the derived equation was evaluated in vivo using a large human cohort (n_2_ = 2263) from the Asklepios study^12^.

## Materials and methods

### In silico dataset

In silico data that were generated in our previous work^10^ were used to simulate various hemodynamic cases. The data generation relied on a previously developed, well-validated one-dimensional cardiovascular computer simulator^11^ which ran using different combinations of physiologically relevant input model parameters. The distributions of the input model parameters were based on literature data, by identifying the normal values and ranges of the parameters. The parameters of arterial distensibility, terminal compliance, and peripheral resistances were altered to achieve the specific value in the selected ranges. Furthermore, the geometry of the arterial network (namely length, inlet diameter, and outlet diameter of the arterial segments) was modified to simulate different body types by adapting the length and the diameter of all arterial vessels. A detailed description of the data generation processes is provided in the original publication^10^.

In silico blood pressure data, such as the mean, systolic and diastolic blood pressure, pulse pressure, at the left common carotid artery and the brachial artery were extracted from the simulations. The ${\uptau }$ values were derived as the product of total peripheral resistance ($R$) and total arterial compliance ($C$). The total arterial compliance of the in silico data was calculated analytically by summing of the incremental volume compliance of all arterial segments.

### In vivo dataset—Asklepios study (round 1)

Human data were made available from baseline (round 1) data of the Asklepios study, a broad prospective longitudinal study with the aim of assessing the development and progression of cardiovascular disease^12^. A total of 2404 subjects were found eligible to be included in the study. The inclusion and exclusion criteria are listed in Table 1. The participants underwent a non-invasive evaluation of central hemodynamics, including recordings of carotid blood pressure. The study protocol was approved by the ethical committee of Ghent University Hospital and informed consent of participation was given by all subjects. All experiments were performed in accordance with relevant guidelines and regulations. A comprehensive description of the Asklepios data can be found in the original publication^12^.

**Table 1:** Asklepios inclusion and exclusion criteria^12^.

**Inclusion criteria**
1. Male and female volunteers aged 35–55 years at study initiation, living in the communities of Erpe–Mere or Nieuwerkerken
**Exclusion criteria**
1. Clinical presence of atherosclerosis/atherothrombosis
(a) Atherosclerosis: symptomatic or haemodynamically significant (> 50% stenosis) presence of atherosclerosis in any major vascular bed
(b) Atherothrombosis: acute coronary syndromes, cerebrovascular thrombosis
(c) Previous or planned revascularization procedure (carotid, coronary, lower limb)
2. Major concomitant illness
(a) Cardiac: cardiomyopathy/heart failure, significant valvular disease, previous cardiac surgery, (complex) congenital heart disease, heart transplant
(b) Organ failure: end-stage renal disease, hepatic insufficiency, previous organ transplant
(c) Malignant tumours (recently diagnosed or currently treated, with < 3 years tumour-free follow-up or tumours that are metastatic or initial treatment was not curative)
(d) Other conditions in which the screening physician expected a life expectancy < 5 years
3. Diabetes mellitus
(a) Diabetes mellitus type 1
(b) Diabetes mellitus type 2 if confirmed macrovasculopathy (see exclusion criterion 1) or significant renal impairment [see exclusion criterion 2(b)]
4. Specific conditions precluding accurate haemodynamic assessment
(a) Continually irregular cardiac cycle: atrial fibrillation
(b) State of hyperdynamic activity: pregnancy (in the preceding 6 months)
5. Inability to provide informed consent

In the original study reporting the Asklepios data (round 1), central blood pressure waveforms were recorded at the left common carotid artery via applanation tonometry using a Millar pen-type tonometer (SPT 301; Millar Instruments, Houston, Texas, USA). The measurement set-up, processing, and calibration procedure (based on sphygmomanometer systolic and diastolic blood pressure and applanation tonometry at the brachial artery) were previously described in detail^12,13^.

The carotid pressure was derived as a “mean” waveform of multiple beats from a 20-s recording^14^. Pressure data were recorded in continuous sequences of 20 s. The Savitsky-Golay filter implemented in Matlab, The Mathworks Inc. was used to post-process the data. Identification of individual cycles, detrending (i.e., linearly smoothing out eventual differences in the numerical value of the start and end of the cycle), and averaging were performed. The average of these cycles was considered as the tonometry recording for the carotid artery. The calibration of the carotid waveform was based on the assumption that diastolic and mean blood pressure values remain fairly constant for the major arteries. The carotid pressure waveforms were calibrated to the diastolic and mean arterial blood pressure, with diastolic blood pressure taken from brachial cuff blood pressure measurement. Mean arterial pressure was assessed following a calibration scheme as the average of a brachial artery applanation tonometry waveform, calibrated to diastolic and systolic cuff blood pressure, following^15^.

The ${\uptau }$ values were derived as the product of total peripheral resistance ($R$) and total arterial compliance ($C$). In the Asklepios data, total arterial compliance was estimated using the pulse pressure method^16^. The pulse pressure method is grounded in the observation that the modulus of the input impedance of the arterial system aligns closely with the two-element Windkessel model, particularly within the low frequencies spanning the 1st to the 5th harmonic. This implies a strong resemblance between the pulse pressure in the actual arterial system and that in the two-element Windkessel model. The method employs an iterative approach, aiming to determine the optimal value of total arterial compliance. This is achieved by iteratively refining total arterial compliance to achieve the best fit between the measured pulse pressure and the pulse pressure predicted by the two-element Windkessel model.

### Analytical derivation of the formulas

In our analysis, we started from the widely accepted formula which suggests that total arterial compliance ($C$) is proportional to stroke volume ($SV$) divided by central pulse pressure ($cPP$)^3,17,18^:

1 $$C \propto \frac{SV}{{cPP}}\,or\,C = k\frac{SV}{{cPP}}$$

In addition, the following equation holds:

2 $$R = \frac{MBP}{{CO}}$$

where MBP is the mean pressure which is the same across all large and mid-sized arteries and $CO$ is the cardiac output which equals heart rate ($HR$) times $SV$, namely:

3 $$CO = SV \cdot HR$$

Finally, $HR$ equals 60/$T$, where $T$ is the time duration of a heartbeat.

Therefore, starting from Eq. (1), we can derive the relationship between $RC$ and $T\frac{MBP}{{cPP}}$ as follows:

4 $$\begin{gathered} C = k\frac{SV}{{cPP}} \Rightarrow RC = Rk\frac{SV}{{cPP}} \;\; \Rightarrow RC = Rk\frac{SV}{{cPP}} \mathop \Rightarrow \limits^{\tau = RC} \tau = kR\frac{SV}{{cPP}} \hfill \\ \mathop \Rightarrow \limits^{{R = \frac{MBP}{{CO}} \left( {Eq.2} \right)}} \tau = k\frac{MBP}{{CO}}\frac{SV}{{cPP}} \mathop \Rightarrow \limits^{{CO = SV \cdot HR \left( {Eq.3} \right)}} \tau = k\frac{MBP}{{SV \cdot HR}}\frac{SV}{{cPP}} \hfill \\ \Rightarrow \tau = k\frac{1}{HR}\frac{MBP}{{cPP}}\mathop \Rightarrow \limits^{HR = 60/T} \tau = k\frac{T}{60}\frac{MBP}{{cPP}} \hfill \\ \end{gathered}$$

5 $$\tau = k^{\prime}T\frac{MBP}{{cPP}}$$

where $$k^{\prime} = \frac{k}{60}$$.

The constant $k^{\prime}$ in Eq. (5) was derived empirically using linear fitting on the in silico data. Subsequently, the resulted formula was evaluated on the in vivo data of the Asklepios cohort. In our analysis, the $cPP$ was set equal to the central (carotid) pulse pressure value.

Moreover, we implemented the same fitting process, that was performed on the in silico population, to derive the precise constant $k^{\prime}$ for the Asklepios population data. The age and gender dependency of the constant $k^{\prime}$ was investigated. Our initial hypothesis was that $k^{\prime}$ varies with age and gender. In this respect, the fitting analysis was performed separately for three different age groups, namely < 40 years, 40–50 years and > 50 years for male and female subjects. Our investigation encompassed simultaneous and separate examinations of age- and gender-specific differences in the $k^{\prime}$ coefficient’s value.

Additionally, we examined potential dependencies between central pulse pressure and heart rate (HR) as well as mean blood pressure (MBP). To investigate whether the coefficient $k^{\prime}$ is influenced by HR and MBP, we split the dataset into three groups according to the distribution of each independent variable (namely HR and MBP) using percentiles. The data were sorted based on the variable of interest, and we established the first percentile group (< Q1), the second percentile group [Q1,Q3], and the third percentile group (> Q3). Each data point was then assigned to a group based on its percentile.

Within the scope of our investigation, we also analyzed the sensitivity of the $k^{\prime}$ coefficient to variations in MBP estimation. We selected various MBP derivation formulas, guided by the recommendations from the study conducted by Papaioannou et al.^25^. The objective was to quantitatively assess the impact of differing MBP estimation formulas on the variability of the $k^{\prime}$ coefficient.

Finally, the formula using the in silico-derived coefficient $k^{\prime}$(from the entire in silico dataset) was applied to make $\tau$ estimations for the Asklepios subjects. The accuracy of the estimated $\tau$ values was assessed by considering gender and age as separate criteria; namely the data were divided into male and female categories, as well as different age groups, to evaluate the accuracy with respect to these demographic factors.

### Statistical analysis

All data are presented as mean and standard deviation (SD). The statistical analysis was performed in Python (Python Software Foundation, Python Language Reference, version 3.11.7, available at http://www.python.org). The correlation, conformity, and precision between the estimations (using the linear formula) and the reference data were evaluated using the Pearson’s correlation coefficient (r), intraclass correlation coefficient (ICC), and the normalized root mean square error (RMSE). The computed normalized RMSE was based on the difference between the minimum and maximum values of the dependent variable (y) and was computed as RMSE/(y_max_−y_min_). Bias and limits of agreement (LoA) (where the 95% of errors are expected to lie) were calculated using the Bland–Altman analysis^19^. Linear least-squares regression was performed for the estimated and reference data. The slope and the intercept of the regression line were reported. Two-sided p-values for hypothesis tests were calculated using Wald Tests with t-distribution of the test statistic. The null hypothesis was that the slope is zero. A p < 0.05 was considered statistically significant.

## Results

Out of the 2404 human participants in the Asklepios study, 141 were excluded due to inaccurate or missing data. The in vivo population consisted of 1090 (48%) male participants and 1173 (52%) female participants. The characteristics of the in silico (n_1_ = 3818) and in vivo (n_2_ = 2263) populations are described in Table 2. Mean and SD values were reported to be similar for brachial blood pressure values between the two populations. Central (carotid) systolic blood pressure (SBP) values were found to be slightly higher in the Asklepios data, whereas central (carotid) diastolic blood pressure (DBP) was overall lower, thus leading to a higher mean pulse pressure in the in vivo human data. In addition, in vivo heart cycle was higher in comparison to the in silico heart cycle values. Similar distributions were observed for total arterial compliance and total peripheral resistance. In vivo cardiac output was reported to be lower when compared to the in silico cardiac output by approximately 18%. The latter is to be expected if we consider that for the same value of mean blood pressure (in silico data: 101 ± 21 mmHg and in vivo data: 100 ± 12 mmHg) and higher total peripheral resistance (in silico data: 1 ± 0.2 mmHg s/mL and in vivo data: 1.3 ± 0.4 mmHg s/mL), the cardiac output is expected to be adjusted accordingly.

**Table 2:** Description of the cardiovascular characteristics and parameters of the in silico and in vivo data.

Parameter	In silico population n_1_ = 3818	In vivo population n_2_ = 2263
	Mean ± SD	Mean ± SD
Age (years)	n/a	46 ± 6
Gender (M/F)	n/a	1090/1173
Height (cm)	n/a	169 ± 9
Weight (kg)	n/a	74 ± 14
Central (carotid) systolic blood pressure (mmHg)	124 ± 23	131 ± 17
Central (carotid) diastolic blood pressure (mmHg)	80 ± 21	77 ± 11
Central (carotid) pulse pressure (mmHg)	45 ± 19	54 ± 12
Mean blood pressure (mmHg)	101 ± 21	100 ± 12
Brachial systolic blood pressure (mmHg)	135 ± 24	132 ± 15
Brachial systolic blood pressure (mmHg)	77 ± 21	77 ± 11
Brachial pulse pressure (mmHg)	57 ± 23	54 ± 10
Cardiac output (L/min)	6 ± 1.2	4.9 ± 1.2
Heart cycle (s)	0.7 ± 0.1	1 ± 0.2
Total arterial compliance (mL/mmHg)	1.1 ± 0.5	1.4 ± 0.5
Total peripheral resistance (mmHg.s/mL)	1 ± 0.2	1.3 ± 0.4
Time constant ${\uptau }$ (s)	1.3 ± 0.7	1.2 ± 0.3

### Derivation of the theoretical ${{\varvec{\uptau}}}$ formula (Eq. 5) using the in silico data

The constant $k^{\prime}$ was defined by fitting the true ${\uptau }$ and the right part of Eq. 5 using the in silico population. By performing linear regression analysis between the true ${\uptau }$ and the product $T\frac{MBP}{{cPP}}$, we derived the mapping equation ${\uptau } = 0.7$ $T\frac{MBP}{{cPP}}$ (R^2^ = 0.9), as shown in Table 3. Table 4 presents the in silico results of the linear fitting performed on the HR- and MBP-dependent groups, indicating insignificant changes in the derived $k^{\prime}$ values. In addition, the analysis of the $k^{\prime}$ coefficient’s sensitivity to the MBP estimation revealed minimal variability when applying different estimation formulas (Table 5). This negligible variation in the $k^{\prime}$ coefficient is attributed to the observation that only slight discrepancies in MBP values were obtained across the various computational techniques.

**Table 3:** Derivation of coefficient $k^{\prime}$ from linear fitting.

	Coefficient $k^{\prime}$		
	All	Male	Female
Linear fitting (in silico data)	n = 3818		
	0.7 (R^2^ = 0.9)	n/a	n/a
Linear fitting (in vivo data—all ages)	n = 2263	n = 1090	n = 1173
	0.64 (R^2^ = 0.88)	0.65 (R^2^ = 0.89)	0.62 (R^2^ = 0.85)
Linear fitting (in vivo data— < 40 years)	n = 456	n = 216	n = 240
	0.64 (R^2^ = 0.86)	0.66 (R^2^ = 0.9)	0.62 (R^2^ = 0.82)
Linear fitting (in vivo data—40–50 years)	n = 1′147	n = 558	n = 589
	0.64 (R^2^ = 0.88)	0.65 (R^2^ = 0.9)	0.62 (R^2^ = 0.86)
Linear fitting (in vivo data— > 50 years)	n = 660	n = 316	n = 344
	0.63 (R^2^ = 0.87)	0.64 (R^2^ = 0.89)	0.61 (R^2^ = 0.84)

**Table 4:** Derivation of coefficient $k^{\prime}$ from linear fitting for different heart rate (HR) and mean blood pressure (MBP) groups.

	Coefficient $k^{\prime}$								
	< Q1_HR_			[Q1_HR_, Q3_HR_]			> Q3_HR_		
	Male and female	Male	Female	Male and female	Male	Female	Male and female	Male	Female
Linear fitting (in silico data)	n = 1273	n/a	n/a	n = 1272	n/a	n/a	n = 1273	n/a	n/a
	0.72 (R^2^ = 0.91)	n/a	n/a	0.7 (R^2^ = 0.9)	n/a	n/a	0.68 (R^2^ = 0.9)	n/a	n/a
Linear fitting (in vivo data^a^)	n = 775	n = 397	n = 372	n = 766	n = 394	n = 360	n = 722	n = 382	n = 358
	0.66 (R^2^ = 0.85)	0.64 (R^2^ = 0.79)	0.67 (R^2^ = 0.88)	0.63 (R^2^ = 0.86)	0.62 (R^2^ = 0.85)	0.65 (R^2^ = 0.88)	0.61 (R^2^ = 0.89)	0.6 (R^2^ = 0.88)	0.62 (R^2^ = 0.9)

	< Q1_MBP_			[Q1_MBP_, Q3_MBP_]			> Q3_MBP_		
	Male and female	Male	Female	Male and female	Male	Female	Male and female	Male	Female
Linear fitting (in silico data)	n = 1273	n/a	n/a	n = 1272	n/a	n/a	n = 1273	n/a	n/a
	0.74 (R^2^ = 0.91)	n/a	n/a	0.71 (R^2^ = 0.92)	n/a	n/a	0.66 (R^2^ = 0.88)	n/a	n/a
Linear fitting (in vivo data^a^)	n = 755	n = 391	n = 364	n = 753	n = 391	n = 363	n = 755	n = 391	n = 363
	0.64 (R^2^ = 0.87)	0.63 (R^2^ = 0.86)	0.66 (R^2^ = 0.89)	0.64 (R^2^ = 0.89)	0.62 (R^2^ = 0.86)	0.65 (R^2^ = 0.91)	0.63 (R^2^ = 0.87)	0.61 (R^2^ = 0.85)	0.64 (R^2^ = 0.89)

^a^Including all age groups.

**Table 5:** Variation in $k^{\prime}$ coefficient with respect to MBP estimation formulas.

	In silico data		In vivo data^a^	
MBP estimation formula	MBP (mmHg) mean ± SD	Linear fitting	MBP (mmHg) mean ± SD	Linear fitting
MBP = average(BP)	101 ± 21	0.7 (R^2^ = 0.9)	100 ± 12	0.64 (R^2^ = 0.88)
MBP = 0.42 × SBP + 0.58 × DBP^20^	101 ± 20	0.7 (R^2^ = 0.91)	100 ± 12	0.63 (R^2^ = 0.88)
MBP = DBP + 0.33 × PP^21^	96 ± 20	0.72 (R^2^ = 0.93)	95 ± 12	0.66 (R^2^ = 0.87)
MBP = DBP + 0.33 × PP + 5^22^	101 ± 20	0.69 (R^2^ = 0.93)	100 ± 12	0.63 (R^2^ = 0.88)
MBP = DBP + [0.33 + (0.0012 × HR)] × PP^23^	102 ± 20	0.69 (R^2^ = 0.91)	99 ± 12	0.64 (R^2^ = 0.87)
MBP = (SBP × DBP)^1⁄2^^24^	101 ± 21	0.69 (R^2^ = 0.91)	101 ± 12	0.63 (R^2^ = 0.88)

^a^Including all age groups.

MBP: mean blood pressure; SBP: systolic blood pressure; DBP: diastolic blood pressure; PP: pulse pressure; HR: heart rate.

### Evaluation of the theoretical ${\varvec{\uptau}}$ formula (Eq. 5) using the in vivo data

The results from the fitting processes for each Asklepios age group are aggregated in Table 3. The results indicate that $k^{\prime}$ does not vary significantly across the age groups. No significant difference in the coefficient’s value was observed for the gender-specific analysis. In addition, the $k^{\prime}$ values yielded from the separate Asklepios groups of HR- and MBP-dependent groups are presented in Table 4. Similarly, no pronounced variation was reported.

**Figure 1:**
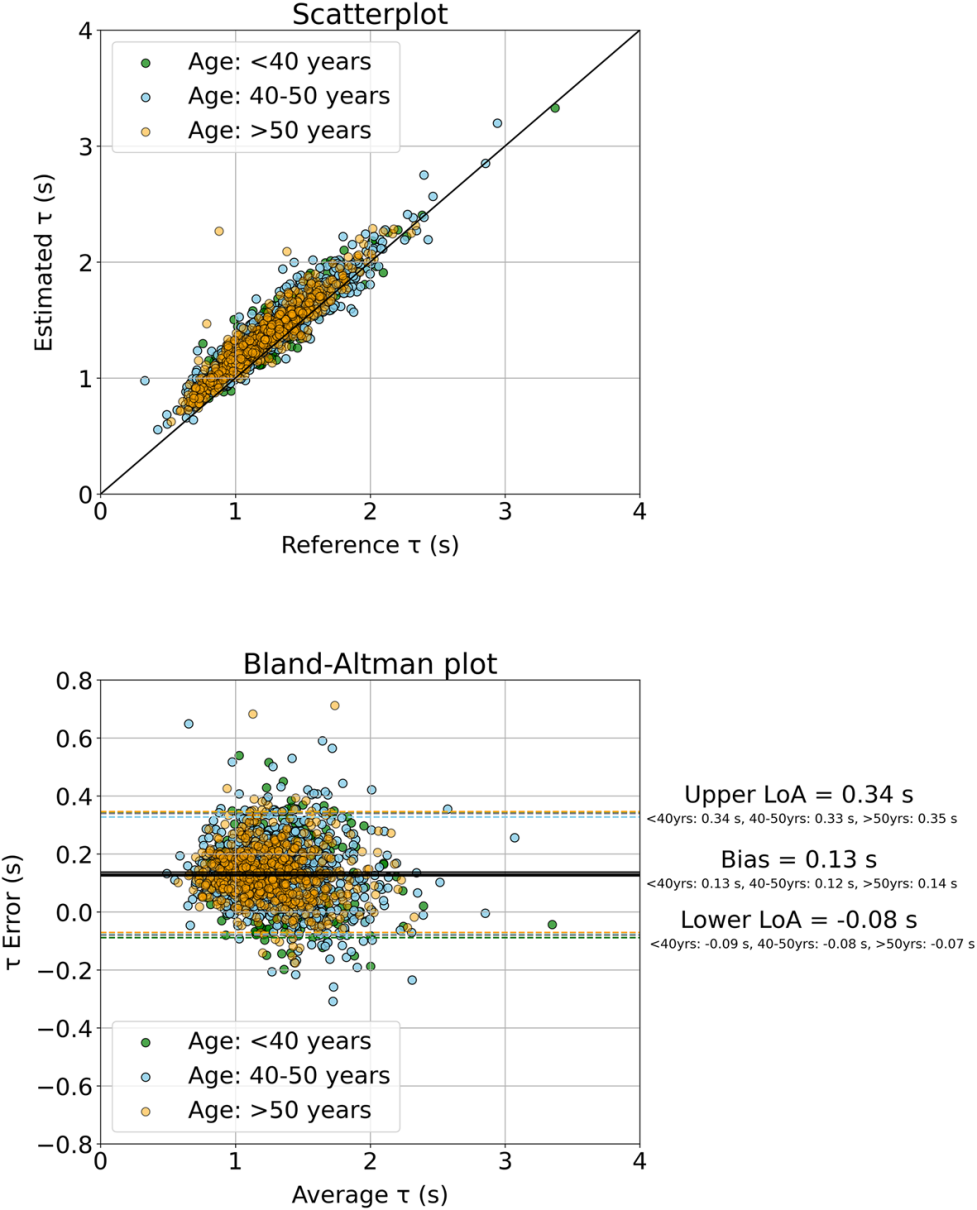
Scatter plot and Bland–Altman analysis between the estimated ${\uptau }$ values using formula ${\uptau } = 0.7T\frac{MBP}{{cPP}}$ and the reference values for the Asklepios data.

Subsequently, the resulted formula $\left[ {{\uptau } = 0.7T\frac{MBP}{{cPP}}} \right]$ was applied on the clinical data from the Asklepios study. The estimated versus the reference ${\uptau }$ data are presented in Fig. 1. A high agreement was observed between the estimated and the reference ${\uptau }$ values, with a Pearson’s correlation coefficient equal to 0.94 across all ages. The slope was found to be 0.9 (p < 0.0001) and the intercept was 0.25 s for all age groups. The Bland–Altman analysis yielded a low bias of 0.13 s and limits of agreement equal to [− 0.08, 0.34] s across all ages, while the normalized RMSE was reported to be low and equal to 5.5%. The age- and gender-specific results are reported in detail in Table 6. The study generally reported a modest overestimation, with the errors being marginally greater (nRMSE exceeding 10%) for female groups of < 40 years and > 50 years compared to the respective male groups.

**Table 6:** Metrics of accuracy, correlation, and agreement between estimated and reference $\uptau$ values for the Asklepios subjects.

Group	r/ICC	Slope	Intercept (s)	nRMSE (%)	Bias (s)	Limits of agreement (s)
All ages (n = 2263)	0.94/0.82	0.9 (p < 0.0001)	0.25	5.5	0.13	[− 0.08, 0.34]
All ages, Male (n = 1090)	0.95/0.84	0.9 (p < 0.0001)	0.24	5.2	0.11	[− 0.09, 0.3]
All ages, Female (n = 1173)	0.92/0.8	0.93 (p < 0.0001)	0.22	8.6	0.15	[− 0.06, 0.36]
Age: < 40 years (n = 456)	0.93/0.8	0.89 (p < 0.0001)	0.27	6.3	0.13	[− 0.09, 0.34]
Age: < 40 years, Male (n = 216)	0.95/0.86	0.9 (p < 0.0001)	0.23	5.1	0.09	[− 0.11, 0.29]
Age: < 40 years, Female (n = 240)	0.91/0.75	0.93 (p < 0.0001)	0.25	12.6	0.16	[− 0.05, 0.37]
Age: 40–50 years, (n = 1147)	0.94/0.82	0.9 (p < 0.0001)	0.25	6.2	0.12	[− 0.08, 0.33]
Age: 40–50 years, Male (n = 558)	0.95/0.85	0.9 (p < 0.0001)	0.24	6.3	0.11	[− 0.09, 0.3]
Age: 40–50 years, Female (n = 589)	0.93/0.8	0.92 (p < 0.0001)	0.24	8.3	0.14	[− 0.06, 0.35]
Age: > 50 years, (n = 660)	0.94/0.82	0.91 (p < 0.0001)	0.24	9.6	0.14	[− 0.07, 0.35]
Age: > 50 years, Male (n = 316)	0.95/0.82	0.9 (p < 0.0001)	0.25	8.7	0.12	[− 0.08, 0.32]
Age: > 50 years, Female (n = 344)	0.92/0.81	0.96 (p < 0.0001)	0.2	13.5	0.15	[-0.06, 0.37]

r, Pearson’s correlation coefficient; ICC, intraclass correlation coefficient; nRMSE, normalized root mean square error.

## Discussion

A new relationship was established between the central diastolic pressure decay time constant, ${\uptau }$, and the duration of the heart cycle and the ratio of mean blood pressure over central pulse pressure. Linear fitting was applied to estimate the linear coefficient using in silico data^10^ which were generated using a validated one-dimensional mathematical model of the cardiovascular system^11^. Validation of the derived equation was performed using in vivo data from the large Asklepios cohort^12^. To our knowledge, this is the first study providing an explicit relationship between central diastolic pressure decay time constant and basic central pressure values. The derived formula was based on the generic $C \propto \frac{SV}{{cPP}}$ equation and appeared to apply well over a wide range of simulated and real physiological conditions.

The value of the linear coefficient was similar in both the synthetic and the human data, while the coefficient was reported to be age- and gender-independent. Notably, the in silico data provided valuable insights into the actual value of the coefficient $k^{\prime}$ in real human data. Although the parameter distributions between the in silico and the in vivo population were not identical (Table 2), the in silico data appeared to simulate well the content of the real human population. These findings constitute another proof highlighting the significance of mathematical modelling in studying, understanding phenomena, but also quantifying physiological relationships between cardiovascular quantities.

Our study demonstrated that linear fitting across HR- and MBP-dependent groups yields $k^{\prime}$ values with insignificant fluctuations, suggesting a uniform response of the $k^{\prime}$ coefficient to variations in HR and MBP levels. Furthermore, our examination of the $k^{\prime}$ coefficient’s sensitivity to MBP estimation methods revealed only minimal variability with different estimation formulas. This slight variation in $k^{\prime}$ values was attributable to the fact that only minor differences were observed in MBP calculations across methods. Importantly, these findings indicate a consistent behavior of the  $k^{\prime}$ coefficient across varying levels of HR and MBP, highlighting its reliability and applicability in diverse physiological conditions.

Previous research has demonstrated that the decay time of diastolic pressure holds significant pathophysiological data from a clinical perspective. Aortic stiffening with reduced compliance may impair myocardial viability by hastening the diastolic exponential decay of the central blood pressure, rather than by increasing late-systolic augmentation. This predisposes hypertensive patients to ischemic heart disease^26^. Providing a feasible substitute for aortic diastolic decay information may enable new strategies in the evaluation of vascular health. In addition, the findings of this study could be relevant in diseases where heart-arterial interactions are important, such as in patients with heart failure with preserved ejection fraction (HFpEF), where additional markers to monitor potential abnormal ventricular filling during diastole might be valuable.

The proposed empirical relationship provides a simple and fast formula to compute diastolic pressure decay time constant (${\uptau }$) using heart period, MBP, and $cPP$ without requiring the entire central pressure waveform. Undoubtedly, central pulse pressure is not as accessible as the brachial pulse pressure value acquired by the conventional cuff. When our analysis was repeated using the brachial (peripheral) PP instead of the carotid (central) PP, the fitting produced the following outcomes: $k^{\prime} = 0.89$ (R^2^ = 0.93) for the in silico data and $k^{\prime} = 0.65$ (R^2^ = 0.71) for the in vivo data, respectively. The variation in the coefficient $k^{\prime}$ could be attributed to the disparity in central-to-peripheral pressure amplification between the two datasets. Yet, the linear relationship remains valid. If this modeling criterion is met or if sufficient in vivo data are available (for experimentally deriving the linear coefficient), the brachial pulse pressure could serve as a viable alternative. Importantly, current advancements in the field of non-invasive central blood pressure monitoring can provide accurate approximations. In a previous study, we demonstrated that machine learning can offer robust and precise means to predict aortic blood pressure from cuff (brachial) systolic and diastolic blood pressure^27^. Moreover, in an inverse approach, having the central diastolic pressure decay time constant available enables the derivation of central pulse pressure, as mean blood pressure can be easily derived in clinical settings (e.g. cuff blood pressure measurement).

A major limitation of the proposed study is that the relationship that maps ${\uptau }$ to the product $T\frac{MBP}{{cPP}}$ was evaluated solely on healthy individuals under physiological conditions. Future work should emphasize on the applicability of the proposed formula in other populations including pathologies and diseases. In addition, as the Asklepios participants are a representative cohort of 35–55-year-old individuals, free from overt cardiovascular disease at study initiation, while only about 10% of the subjects (259) received anti-hypertensive medication. The size of this cohort is small for a meaningful sub-analysis according to different medication types. Yet, exploring relationships in reference to specific medications such as beta-blockers or renin–angiotensin–aldosterone system (RAAS) agents may be considered in future research endeavors. Finally, while our study provides valuable insights into the Asklepios population within a specific age range, it is important to acknowledge the limitation of lacking data for individuals aged < 35 and > 55 years. This recognition is particularly significant given the prevalence of cardiovascular disease in the latter age group.

It should also be noted that, in this study, non-invasive carotid blood pressure waveforms were used as a surrogate of central blood pressure, as invasive aortic blood pressure data are difficult to acquire in vivo, especially in large quantity. However, carotid blood pressure is considered as a well-established surrogate of central blood pressure, and it is frequently used as a replacement to the aortic blood pressure measurement. Adding to this, recent evidence demonstrated a near-absolute agreement between the aortic and the carotid ${\uptau }$ values in an in silico population of virtual subjects^28^, contributing additional support to the similarities observed in the pressure waves between the aorta and the carotid.

## Conclusion

This study provides innovative theoretical insights into the association between ${\uptau }$ and central blood pressure features. The presented empirical formula may facilitate the derivation of ${\uptau }$ without the necessity of obtaining the entire aortic blood pressure wave, particularly when an approximation of central pulse pressure is viable (e.g. transformation of peripheral pulse pressure to central pulse pressure). Additionally, this study contributes to existing literature by enhancing the accessibility of an additional biomarker, such as the central diastolic pressure decay time constant (${\uptau }$), potentially opening new avenues for assessing vascular aging or vascular risk factors.

## Data Availability

The in silico dataset used and analysed in the current study is available from the corresponding author (vickybikia@gmail.com) on reasonable request. The in vivo data that support the findings of this study are available from the University of Ghent but restrictions apply to the availability of these data, which were used under license for the current study, and so are not publicly available. Data are however available from the authors upon reasonable request and with permission of the University of Ghent (Patrick.Segers@UGent.be).
